# Primary healthcare practitioners and patient blood management in Africa in the time of coronavirus disease 2019: Safeguarding the blood supply

**DOI:** 10.4102/phcfm.v12i1.2457

**Published:** 2020-05-21

**Authors:** Claire L. Barrett

**Affiliations:** 1 School of Clinical Medicine, Faculty of Health Sciences, University of the Free State, Bloemfontein, South Africa

**Keywords:** Blood supply, patient blood management, Africa, COVID-19, esilience, transfusion

## Abstract

The coronavirus disease 2019 (COVID-19) pandemic has highlighted various weaknesses in global healthcare services. The blood supply in Africa is a critical element of the healthcare service that may be significantly affected by the pandemic. By implementing principles of patient blood management, primary healthcare practitioners may play an important role in the resilience of the blood supply during the COVID-19 pandemic.

## Resilience, the pandemic and the blood supply in Africa

Tomorrow belongs to the people who prepare for it today. (African Proverb)

The World Health Organization (WHO) defines resilience in the context of the provision of essential health and health-related services as ‘the inbuilt capacity of the system to sustain provision of essential health and health-related services even when challenged by outbreaks, disasters or other shocks’.^1^ The coronavirus disease 2019 (COVID-19) pandemic will undoubtedly test the global resilience of the blood supply, and significantly so in Africa. The primary healthcare practitioner plays a crucial role in reducing the burden of anaemia, thus safeguarding the blood supply in Africa.

The COVID-19 pandemic showcases weaknesses, shortcomings and lack of resilience in global healthcare services. Whilst commendable work has been performed in health disaster risk management in Africa,^2^ and recommendations made on how to maintain the blood supply during infectious outbreaks and the COVID-19 pandemic,^3,4^ no recommendation can entirely safeguard the blood supply. Many countries have well-established healthcare systems and access safe blood, yet this is not true for most of Africa. Although the demand for blood is high, blood donation rates are very low, especially in low and lower middle-income countries in Africa.^1^ A third of maternal deaths in sub-Saharan Africa are because of maternal haemorrhage, and this region has the highest maternal mortality in the world. Access to blood may have prevented up to a quarter of these deaths.^5,6^ In spite of this, many countries in Africa collect less than 10 donations per 1000 population, the target recommended by the WHO.^7^ Twenty-two African countries depend on family, replacement or paid donors, and these donations account for more than 50% of the blood supply.^8^ These challenges, although important, are unlikely to be resolved in the midst of the pandemic. Blood donation may well be further reduced because of donor illness, countries imposing travel restrictions and donor fear of contracting the virus by visiting donor centres. The WHO has published guidelines to ensure the safety of blood donors and staff during the pandemic,^4^ and donors should be reassured that they are unlikely to contract the novel coronavirus by donating blood when correct procedures are followed.

Road safety has improved as a result of COVID-19 because of enforced travel restrictions, which has translated to fewer road traffic accidents (RTAs) in South Africa^9,10^ and abroad.^11^ Although little data are available on the indications for the use of blood and blood products in Africa, infectious diseases, obstetric haemorrhage, sickle cell disease and the broad term *anaemia* are the most common indications for transfusion.^12^ In South Africa, relatively few blood products are issued for general surgery and trauma, (11.3% and 2.8%, respectively), and the bulk of blood products are issued to medical patients, obstetrics and gynaecology and intensive care units (28.9%, 16.9% and 16.7%, respectively),^13^ which is in agreement with previous observation.^12^ These data suggest that even though lockdown may reduce the number of RTAs, the need for blood will persist. This emphasises the need for a sustained blood supply that relies on uninterrupted blood donation, component production, appropriate clinical use of blood and blood products, as well as the implementation of patient blood management (PBM) programmes to alleviate anticipated shortages of donor blood.^14^

## Patient blood management

Implementation of the principles of PBM may prove to be a vital step in maintaining a sustainable blood supply in Africa in the face of the pandemic. Patient blood management is defined by the WHO as ‘a patient-focussed, evidence-based and systematic approach to optimise the management of patient and transfusion of blood products for quality and effective patient care’.^15^ In addition, the WHO emphasises that PBM should minimise unnecessary exposure to blood products and, through health promotion and screening, prevent conditions that may result in the need for transfusion.^15^ Patient blood management is built on three pillars: optimisation of erythropoiesis, minimisation of blood loss, and bleeding and harnessing and optimising physiological reserve of anaemia.^16^ Whilst PBM has been shown to be reduce the need for transfusion, reduce costs and improve patient safety and clinical outcomes, the implementation thereof has lagged behind.^14,17,18^ For the most part, where PBM has been adopted, it has been incorporated into the practice of anaesthetists, surgeons, physicians, intensivists, and obstetricians and gynaecologists. In these disciplines, the focus has been on the identification and management of anaemia and bleeding risk, appropriate and conservative use of blood products and alternatives to transfusion. These are the key elements of PBM; however, conservative transfusion triggers are the norm in most of Africa, and the traditional approach to PBM may only aid a minority of patients who have access to hospital and specialist care. In spite of efforts by the WHO, the burden of anaemia is high in Africa, particularly East sub-Saharan Africa. Iron deficiency, malaria, schistosomiasis, hookworm, sickle cell disease and thalassemia are the main causes of anaemia in Africa.^19^ Many of these conditions are managed by the primary healthcare practitioner, who should thus play a pivotal part in the implementation of PBM in the outpatient setting. Table 1 is adapted from the three-pillar approach from Isbister and Spahn,^16,20^ reworked for use by primary healthcare practitioners in the outpatient setting in Africa.

**TABLE 1:** Three pillars of patient blood management relevant to the primary healthcare practitioner in the resource-limited setting.

First pillar	Second pillar	Third pillar
**Optimise erythropoiesis** Detect anaemia (including well-compensated anaemia) Flag, trace and treat patients with anaemia Identify underlying causes of anaemia Consider common causes of anaemia: nutritional deficiencies (e.g. iron, vitamin B12 and folate deficiencies), malaria, schistosomiasis, hookworm, sickle cell disease, thalassemia and others Ensure early and adequate treatment of anaemia, with patient education on the importance of compliance Intravenous iron may be indicated in pregnant women with iron-deficiency anaemia and other patient groups Identify and avoid medications (including TCAMs) that can decrease erythropoiesis	**Minimise blood loss and bleeding** Identify and manage bleeding risk (detailed personal and family history and clinical examination) Patients with suspected inherited bleeding disorders should be screened using an appropriate tool (ISTH-BAT)^21^ Identify and avoid medications (including TCAMs) that can increase bleeding risk Minimise phlebotomy	**Plan and optimise physiological reserve of anaemia** Identify high-risk patient groups (pregnant women, children and the elderly) Assess and optimise patient’s physiological reserve to anaemia Early identification of patients who may need transfusion Formulate specific management plans for patients who have coagulopathies or may require transfusions Timely identification of patients who need specialist referral Avoid and treat infections promptly

*Source*: Adapted from Isbister^16^ and Spahn and Goodnough^20^

ISTH-BAT, International Society on Thrombosis and Haemostasis Bleeding Assessment Tool; TCAM, traditional, complementary and alternative medications.

These recommendations are contextualised in light of the pandemic and can be applied to all patients who access WHO-compliant priority services, including the care of pregnant women, and patients who access care for emergency conditions, vaccination and the acceptable auxiliary services.^22^ Whilst it is acknowledged that transfusion cannot be avoided in patients with certain conditions and that many patients will still require transfusion as part of their standard care, the application of these principles may reduce the number of transfusions a potential transfusion recipient would need, and may thereby safeguard the blood supply for those who need it most. The ‘common-sense’ principles that underpin PBM have been recommended to address regional and national shortages of blood during the pandemic.^14^

## Conclusion

Primary healthcare practitioners may play an important role in the resilience of the blood supply during the COVID-19 pandemic. The principles of PBM outlined in this article are inexpensive and relatively easy to implement. If these principles are applied to all patients who receive primary healthcare during the pandemic, the blood supply may be safeguarded for those who need it most.
